# Resveratrol Alleviates the Inhibitory Effect of Tunicamycin-Induced Endoplasmic Reticulum Stress on Expression of Genes Involved in Thyroid Hormone Synthesis in FRTL-5 Thyrocytes

**DOI:** 10.3390/ijms22094373

**Published:** 2021-04-22

**Authors:** Gaiping Wen, Klaus Eder, Robert Ringseis

**Affiliations:** Institute of Animal Nutrition and Nutrition Physiology, Justus-Liebig-University Giessen, Heinrich-Buff-Ring 26-32, 35392 Giessen, Germany; gaiping.wen@ernaehrung.uni-giessen.de (G.W.); klaus.eder@ernaehrung.uni-giessen.de (K.E.)

**Keywords:** resveratrol, FRTL-5, thyroid, iodide uptake, NIS, endoplasmic reticulum stress

## Abstract

Recently, ER stress induced by tunicamycin (TM) was reported to inhibit the expression of key genes involved in thyroid hormone synthesis, such as sodium/iodide symporter (NIS), thyroid peroxidase (TPO) and thyroglobulin (TG), and their regulators such as thyrotropin receptor (TSHR), thyroid transcription factor-1 (TTF-1), thyroid transcription factor-2 (TTF-2) and paired box gene 8 (PAX-8), in FRTL-5 thyrocytes. The present study tested the hypothesis that resveratrol (RSV) alleviates this effect of TM in FRTL-5 cells. While treatment of FRTL-5 cells with TM alone (0.1 µg/mL) for 48 h strongly induced the ER stress-sensitive genes heat shock protein family A member 5 (HSPA5) and DNA damage inducible transcript 3 (DDIT3) and repressed NIS, TPO, TG, TSHR, TTF-1, TTF-2 and PAX-8, combined treatment with TM (0.1 µg/mL) and RSV (10 µM) for 48 h attenuated this effect of TM. In conclusion, RSV alleviates TM-induced ER stress and attenuates the strong impairment of expression of genes involved in thyroid hormone synthesis and their regulators in FRTL-5 thyrocytes exposed to TM-induced ER stress. Thus, RSV may be useful for the treatment of specific thyroid disorders, provided that strategies with improved oral bioavailability of RSV are applied.

## 1. Introduction

The endoplasmic reticulum (ER) plays a pivotal role in the synthesis, folding and maturation of more than one-third of all newly synthesized proteins and almost all secretory proteins in the cell [1]. Following co-translational transport into the ER lumen, these proteins must be folded into their correct shape and modified by ER-resident enzymes, like chaperones and glycosylating enzymes [2]. Despite these efforts of the ER-resident enzymes, at least one-third of the proteins transported into the ER lumen are either incompletely or incorrectly folded. When the accumulation of misfolded proteins in the ER lumen exceeds a critical level, the cell is in a state called ER stress. In order to re-establish protein homeostasis and to decrease ER accumulation of misfolded proteins, mammalian cells are equipped with a defence system, called unfolded protein response (UPR) [3,4]. The key mechanisms of the UPR comprise eukaryotic translation initiation factor 2 alpha kinase 3 (EIF2AK3 alias PERK)-dependent transient attenuation of new protein synthesis leading to a decrease of newly synthesized proteins entering the ER (endoplasmic reticulum) to nucleus signaling 1 (ERN1 alias IRE1)-dependent mRNA degradation decreasing the amount of proteins entering the ER co-translationally, and unconventional mRNA splicing of X-box binding protein 1 (XBP1) causing the release of active sXBP1, which induces genes involved in chaperoning, disulfide isomerization, *N*-glycosylation and vesicular trafficking [5].

Cells synthesizing mainly secretory proteins, such as thyrocytes being responsible for the production and secretion of thyroid hormones, have been shown to be highly susceptible to the development of ER stress and, as a consequence, exhibit an impaired secretory function during ER stress [6,7]. In fact, induction of ER stress/UPR has been observed in different thyroid disorders associated with hypothyroidism [7,8,9,10,11]. In line with this, we have recently demonstrated that ER stress induced by tunicamycin (TM)—a compound causing ER stress via inhibiting protein glycosylation [12]—inhibits the expression of the key genes involved in thyroid hormone synthesis, such as sodium/iodide symporter (NIS), thyroid peroxidase (TPO) and thyroglobulin (TG), in FRTL-5 thyrocytes [13]. Moreover, we demonstrated that ER stress decreases the expression of thyrotropin receptor (TSHR), thyroid transcription factor-1 (TTF-1), thyroid transcription factor-2 (TTF-2) and paired box gene 8 (PAX-8), and reduces NIS-mediated iodide uptake, which is an essential step in thyroid hormone synthesis, in FRTL-5 thyrocytes [13]. TSHR, TTF-1, TTF-2 and PAX-8 have been shown to be critically involved in transcriptional regulation of *NIS*, *TPO* and *TG* [14,15,16,17,18]. These findings indicate that ER stress decreases thyroid hormone synthesis through impaired TSH/TSHR signalling in concert with reduced expression of TTF-1, TTF-2, PAX-8. Besides deciphering a potential pathophysiological role of ER stress in thyroid disorders associated with hypothyroidism, these findings may open up therapeutic options in the treatment of thyroid disorders via targeting ER stress.

In recent years, a large number of plant-derived bioactive compounds have emerged as powerful agents for the prevention and treatment of various chronic human diseases. Amongst these compounds, the polyphenol resveratrol (RSV; IUPAC name: 5-[(E)-2-(4-hydroxyphenyl)ethenyl] benzene-1,3-diol), which is naturally found at high concentrations in red grapes and different berries, like blueberries, mulberries and raspberries, is a potential candidate for the treatment of ER stress-associated thyroid disorders. RSV was shown to inhibit ER stress in different cell types and tissues [19,20,21,22,23,24] and to induce the expression of NIS and key regulators of genes involved in thyroid hormone synthesis, such as TTF-1, TTF-2 and PAX-8, in non-transformed FRTL-5 thyrocytes and thyroid carcinoma cells [25,26]. In view of this, the present study tested the hypothesis that RSV alleviates the inhibitory effect of TM-induced ER stress on expression of genes involved in thyroid hormone synthesis and their transcriptional regulators in FRTL-5 thyrocytes.

## 2. Results

### 2.1. Effect of RSV and TM on FRTL-5 Cell Viability

**Figure 1:**
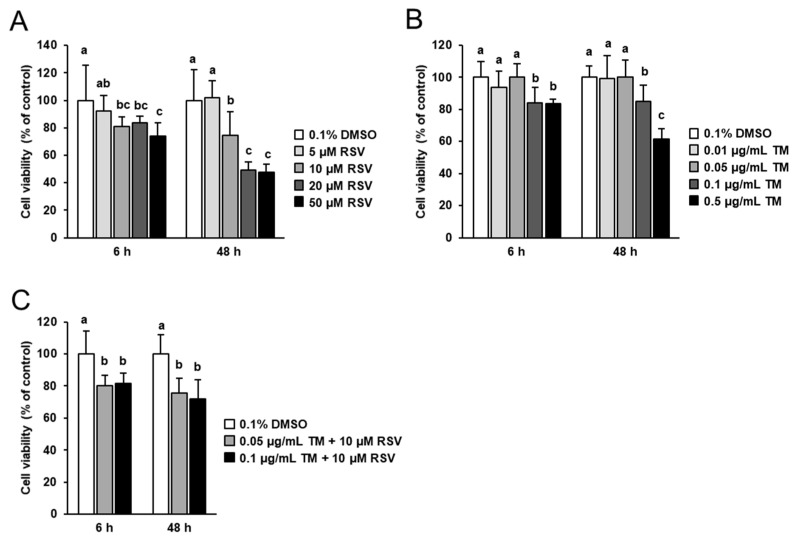
Effect of resveratrol (RSV), tunicamycin (TM) and TM and RSV on FRTL-5 cell viability. After reaching confluence, FRTL-5 cells were treated with different concentrations of either RSV (**A**), TM (**B**) or TM and RSV (**C**) in 6H medium for 6 h and 48 h. Control cells were treated with the same vehicle concentration (0.1% DMSO). Data are means and SD from one independent experiment performed in octuplicate. MTT assay was performed twice and both independent experiments showed similar results. Bars without the same letters (^a,b,c^) within one incubation period differ (*p* < 0.05).

Prior to studying the modulatory potential of RSV on TM-induced ER stress and TM-induced effects on the expression of genes involved in thyroid hormone synthesis, the effects of 6 and 48 h treatment with increasing concentrations of RSV (5 to 50 µM) and TM (0.01 to 0.5 µg/mL) on FRTL-5 cell viability was tested. Treatment of cells with RSV for 6 and 48 h decreased FRTL-5 cell viability at concentrations ≥ 10 µM (Figure 1A). At 10 µM RSV, the decrease of cell viability was 19% and 25% after 6 and 48 h, respectively, compared to control cells. At 20 and 50 µM RSV, cell viability was decreased about 20–25% after 6 h and about 50% after 48 h compared to control cells. Treatment with increasing concentrations of TM for 6 and 48 h did not affect cell viability up to a concentration of 0.05 µg/mL, but decreased cell viability at concentrations ≥ 0.1 µg/mL TM (Figure 1B). The decrease of FRTL-5 cell viability at 0.1 µg/mL TM was approximately 18% after 6 and 48 h of treatment compared to control cells. At 0.5 µg/mL TM, cell viability was decreased about 20% after 6 h and about 40% after 48 h compared to control cells.

For subsequent experiments investigating the modulatory potential of RSV on TM-induced effects, RSV was used at a concentration of 10 µM which was reported to be efficacious in other cell culture studies with FRTL-5 cells [27,28], but did not severely impair FRTL-5 cell viability. TM was used at a concentration of 0.1 µg/mL, because this TM dose was recently shown to clearly induce ER stress in FRTL-5 cells, while only moderately impairing cell viability. In line with this, combined treatment with RSV (10 µM) and TM (0.1 µg/mL) decreased cell viability by 20% after 6 h and 25% after 48 h of treatment (Figure 1C).

### 2.2. Effect of RSV on TM-Induced ER Stress in FRTL-5 Cells

**Figure 2:**
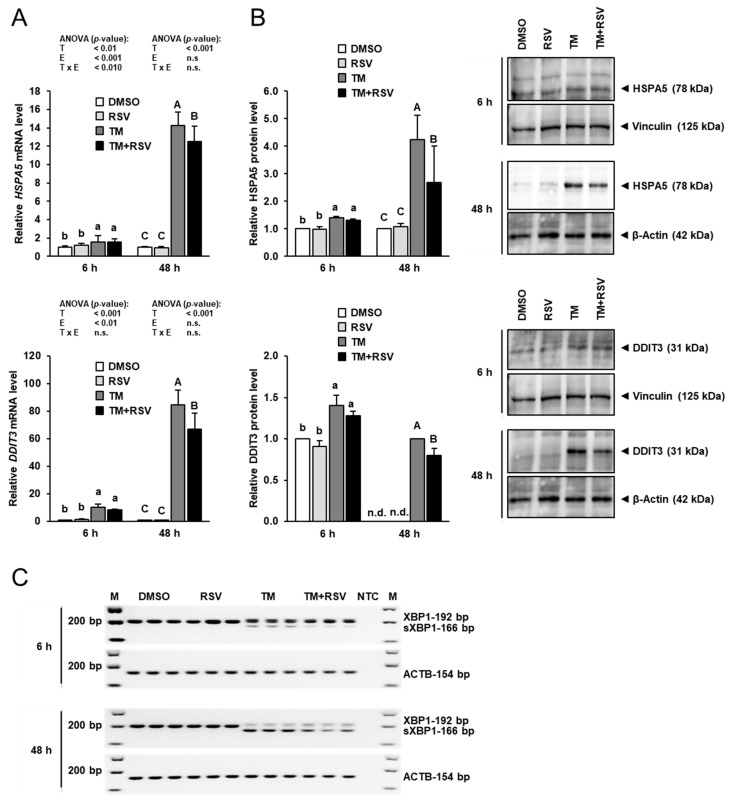
Effect of resveratrol (RSV), tunicamycin (TM) and TM and RSV on markers of ER stress in FRTL-5 cells. After reaching confluence, cells were treated with either 0.1% DMSO (control), 10 µM RSV, 0.1 µg/mL TM or 10 µM RSV and 0.1 µg/mL TM in 6H medium for 6 and 48 h. (**A**,**B**) Relative mRNA levels (**A**) and protein levels (**B**) of HSPA5 and DDIT3 are expressed as fold of control. (**B**) Representative immunoblots for HSPA5 and DDIT3 including immunoblots for β-Actin and Vinculin as internal controls are shown on the right. (**A**) Data from qPCR are means and SD calculated from three replicates for the same treatment of three independent experiments and were subjected to 2-factorial (T = treatment, E = experiment) ANOVA. (**B**) Data from immunoblotting are means and SD calculated from one replicate for the same treatment of three independent experiments and were subjected to 1-factorial ANOVA. Bars without the same letters (^a,b,c, or A,B,C^) differ (*p* < 0.05). (**C**) Representative image from agarose gel electrophoresis of unspliced (192 bp PCR product) and spliced (s) XBP1 (166 bp PCR product) as detected by conventional PCR. The mRNA expression of ACTB served as an internal control. Abbreviation: n.d., not detectable.

To evaluate ER stress, mRNA and protein expression of two ER stress target genes, heat shock protein family A member 5 (HSPA5, also known as BiP) and DNA damage inducible transcript 3 (DDIT3, also known as CHOP), and ER stress-dependent splicing of XBP1 was examined. As shown in Figure 2A, the mRNA levels of *HSPA5* and *DDIT3* were elevated by incubation of FRTL-5 cells with TM (0.1 µg/mL) strongly after 48 h and to a lesser extent after 6 h compared to vehicle control cells. Incubation with RSV (10 µM) alone for 6 and 48 h had no effect on the mRNA levels of *HSPA5* and *DDIT3*. Combined incubation of FRTL-5 cells with RSV and TM for 48 h, but not for 6 h, decreased mRNA levels of *HSPA5* and *DDIT3* by 15–20% compared to FRTL-5 cells incubated with TM alone.

Similar effects were seen at the protein level, as shown in Figure 2B. While treatment of FRTL-5 cells with RSV alone had no effect on protein levels of HSPA5 and DDIT3, treatment with TM alone increased protein levels of HSPA5 and DDIT3 markedly after 48 h and to a lesser extent after 6 h compared to treatment with vehicle. Combined treatment with TM and RSV for 48 h, but not for 6 h, decreased protein levels of HSPA5 and DDIT3 by 35% and 20%, respectively, compared to treatment with TM alone. In line with the effects on HSPA5 and DDIT3 expression, incubation of FRTL-5 cells with TM alone for 6 and 48 h caused XBP1 splicing, a known effect of ER stress, as evident from the detection of a 166 bp PCR product representing sXBP1 (Figure 2C). The band intensity of this sXBP1-specific PCR product was stronger after 48 h than after 6 h, whereas the band intensity of the 192 bp-PCR product representing the unspliced XBP1 was lower after 48 h than after 6 h, which is indicative of the occurrence of stronger ER stress after 48 h. In FRTL-5 cells co-treated with RSV and TM for 6 and 48 h, the band intensity of the sXBP1-specific PCR product was lower than in cells treated with TM alone. Although the 192 bp-PCR product appeared to be marginally increased by RSV in FRTL-5 cells exposed to TM, this effect should not be overinterpreted, because semiquantitative PCR does not allow accurate quantitation and the 192 bp PCR product showed only a very low abundance as indicated by the bright band. Nevertheless, these data clearly show that treatment of FRTL-5 cells with RSV for 48 h attenuates TM-induced ER stress.

### 2.3. RSV Alleviates the Inhibitory Effect of TM-Induced ER Stress on Expression of Genes Involved in Thyroid Hormone Synthesis in FRTL-5 Thyrocytes

**Figure 3:**
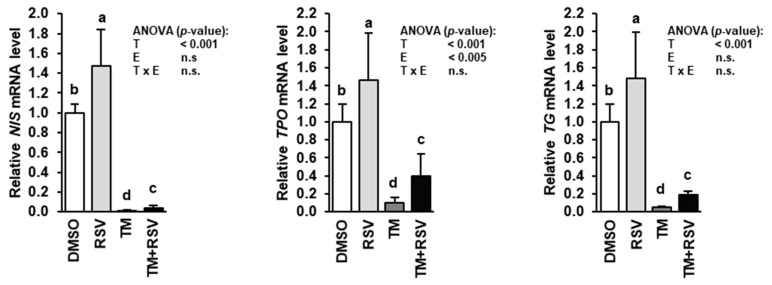
Effect of resveratrol (RSV), tunicamycin (TM) and TM and RSV on mRNA levels of genes involved in thyroid hormone synthesis in FRTL-5 cells. After reaching confluence, cells were treated with either 0.1% DMSO (control), 10 µM RSV, 0.1 µg/mL TM or 10 µM RSV and 0.1 µg/mL TM in 6H medium for 48 h. Relative mRNA levels of *NIS*, *TPO* and *TG* are expressed as fold of control. Data are means and SD calculated from three replicates for the same treatment of three independent experiments and were subjected to 2-factorial (T = treatment, E = experiment) ANOVA. Bars without the same letters (^a,b,c,d^) differ (*p* < 0.05).

Treatment of FRTL-5 cells with RSV alone for 48 h caused an approximately 50% increase of the mRNA levels of *NIS*, *TPO* and *TG* compared to treatment with vehicle (Figure 3), whereas treatment with TM alone for 48 h caused a dramatic reduction of the mRNA levels of *NIS*, *TPO* and *TG* compared to treatment with vehicle. In contrast, in FRTL-5 cells treated with RSV and TM the mRNA levels of *NIS*, *TPO* and *TG* were also markedly lower than in vehicle control cells, but were three to four times higher than in cells treated with TM alone indicating that RSV attenuates the inhibitory effect TM-induced ER stress on *NIS*, *TPO* and *TG* gene transcription.

**Figure 4:**
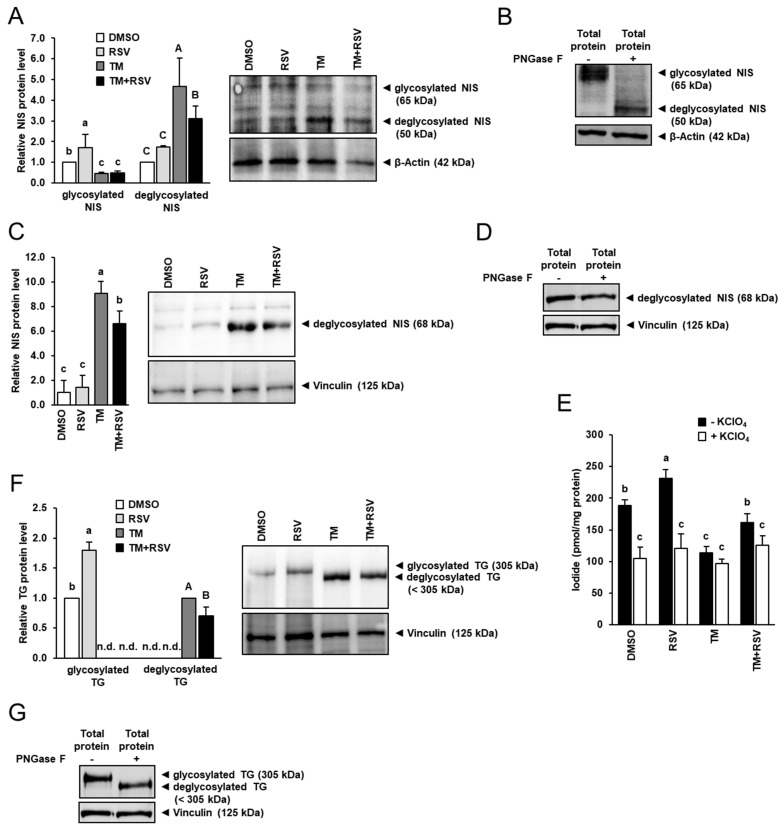
Effect of resveratrol (RSV), tunicamycin (TM) and TM and RSV on protein levels of genes involved in thyroid hormone synthesis and iodide uptake in FRTL-5 cells. After reaching confluence, cells were treated with either 0.1% DMSO (control), 10 µM RSV, 0.1 µg/mL TM or 10 µM RSV and 0.1 µg/mL TM in 6H medium for 48 h. (**A**,**C**,**F**) Relative protein levels of glycosylated and deglycosylated NIS (**A**), deglycosylated NIS (**C**) and glycosylated and deglycosylated TG (**F**) are expressed as fold of control. Representative immunoblots including immunoblots for β-Actin and Vinculin as internal controls are shown. Data from immunoblotting are means and SD calculated from one replicate for the same treatment of three independent experiments and were subjected to 1-factorial ANOVA. (**B**,**D**,**G**) Immunoblots for glycosylated and deglycosylated NIS (**B**), deglycosylated NIS (**D**) and glycosylated and deglycosylated TG (**G**) following treatment of cell protein without (−) or with (+) Peptide-N-Glycosidase F (PNGase F). (**E**) Iodide uptake determined in the absence (−) and presence (+) of the NIS inhibitor KClO_4_. Data are expressed as pmol iodide/mg protein and are means and SD from one independent experiment performed in triplicate. (**A**,**C**,**E**,**F**) Bars without the same letters (^a,b,c, or A,B,C^) differ (*p* < 0.05). Abbreviation: n.d., not detectable.

Both, NIS and TG function are closely regulated by post-translational glycosylation, and the function of both glycoproteins is glycosylation-dependent. In line with this, FRTL-5 cells treated with TM alone showed a reduced protein level of glycosylated NIS (≈65 kDa), but a markedly increased level of dysfunctional deglycosylated NIS (≈50 kDa) compared with vehicle control cells, whereas treatment with RSV alone increased protein level of glycosylated NIS (Figure 4A). The glycosylated and deglycosylated NIS protein could be clearly identified when immunoblotting was carried out with FRTL-5 cell protein treated without (−) and with PNGase F (+), which removes *N*-linked oligosaccharides from glycoproteins (Figure 4B). Accordingly, the band representing the glycosylated NIS was seen only with FRTL-5 cell protein treated without PNGase F, whereas the band representing the low-molecular weight deglycosylated NIS was seen only with FRTL-5 cell protein treated with PNGase F (Figure 4B). In FRTL-5 cells co-treated with RSV and TM, the protein level of glycosylated NIS was also markedly lower than in vehicle control cells but did not differ from cells treated with TM alone (Figure 4A). However, the protein level of deglycosylated NIS was lower in cells co-treated with RSV and TM than in cells treated with TM only, which suggests that RSV decreases the level of dysfunctional deglycosylated NIS under conditions of ER stress. This result was confirmed when immunoblotting was carried out with a NIS-specific antibody against deglycosylated NIS. Figure 4C shows that protein level of deglycosylated NIS was markedly increased by treatment with TM, whereas treatment with RSV in the presence of TM decreased the protein level of deglycosylated NIS by approximately 30%, -an effect that was stronger as seen with the NIS antibody used in Figure 4A. Likely, the weaker contrast between deglycosylated NIS-specific band and background is responsible for the less strong RSV effect observed in Figure 4A. Detection of deglycosylated NIS was confirmed using FRTL-5 cell protein treated without and with PNGase F (Figure 4D).

To investigate whether the altered protein level of deglycosylated NIS was accompanied by an altered NIS function, the effect of RSV and TM was studied on iodide uptake in FRTL-5 cells. Treatment with RSV alone increased total iodide uptake (absence of KClO_4_) by approximately 25%, whereas treatment with TM alone decreased total iodide uptake by approximately 40% compared to control treatment (Figure 4E). Combined treatment with RSV and TM increased total iodide uptake by approximately 40% compared to treatment with TM alone. In the presence of KClO_4_ in the culture medium, treatment with neither RSV nor TM had an effect on iodide uptake indicating that RSV and TM affects NIS-specific iodide uptake.

Like NIS, TG following synthesis becomes glycosylated in the ER of the thyrocyte through addition of *N*-linked oligosaccharide chains. Owing to inhibition of glycosylation by TM, immunoblotting with a TG-specific antibody detecting both glycosylated TG (≈305 kDa, high molecular weight TG) and unglycosylated TG (<305 kDa, low molecular weight TG) revealed the detection of only the low molecular weight TG protein in cells treated with TM alone, whereas the high molecular weight TG protein could not be detected (Figure 4F). Identification of both, glycosylated and deglycosylated TG protein could be confirmed by immunoblotting performed with the same TG antibody for FRTL-5 cell protein treated without and with PNGase F (Figure 4G). In contrast, in cells treated with either vehicle or RSV, only the high molecular weight TG protein could be detected whose expression was markedly increased by treatment with RSV compared to treatment with vehicle. In cells co-incubated with RSV and TM, only the low molecular weight TG was found and its expression was lower than in cells treated with TM only. This indicated that RSV alleviates the inhibitory effect of TM on post-translational protein glycosylation during ER stress.

### 2.4. RSV Alleviates the Inhibitory Effect of TM-Induced ER Stress on Expression of Key Regulators of Thyroid Hormone Synthesis in FRTL-5 Thyrocytes

**Figure 5:**
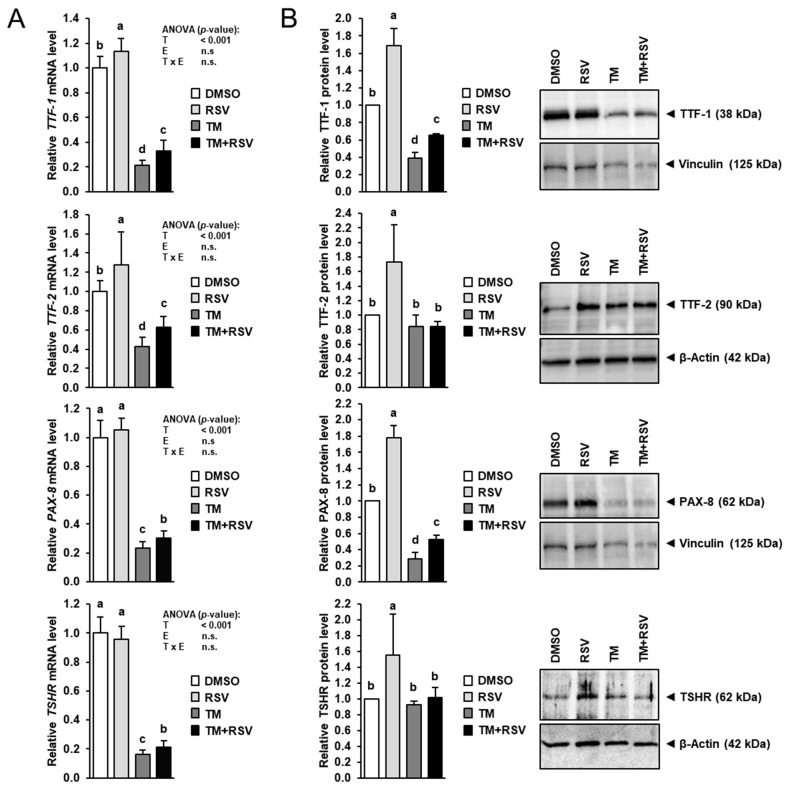
Effect of resveratrol (RSV), tunicamycin (TM) and TM and RSV on expression of genes involved in transcriptional regulation of thyroid hormone synthesizing genes in FRTL-5 cells. After reaching confluence, cells were treated with either 0.1% DMSO (control), 10 µM RSV, 0.1 µg/mL TM or 10 µM RSV and 0.1 µg/mL TM in 6H medium for 48 h. (**A**,**B**) Relative mRNA levels (**A**) and protein levels (**B**) of TTF-1, TTF-2, PAX-8 and TSHR are expressed as fold of control. Representative immunoblots for TTF-1, TTF-2, PAX-8 and TSHR including immunoblots for β-Actin and Vinculin as internal controls are shown on the right. Data from qPCR are means and SD calculated from three replicates for the same treatment of three independent experiments and were subjected to 2-factorial (T = treatment, E = experiment) ANOVA. Data from immunoblotting are means and SD calculated from one replicate for the same treatment of three independent experiments and were subjected to 1-factorial ANOVA. Bars without the same letters (^a,b,c,d^) differ (*p* < 0.05).

To study whether RSV also affects the expression of key regulators of thyroid hormone synthesis, the mRNA and protein levels of TTF-1, TTF-2, PAX-8 and TSHR were determined in FRTL-5 cells treated with RSV in the absence and presence of TM for 48 h. Treatment with RSV alone increased the mRNA levels of *TTF-1* and *TTF-2* by 15–30% compared to treatment with vehicle (Figure 5A), whereas the mRNA levels of *PAX-8* and *TSHR* were not affected. While treatment with TM alone markedly decreased mRNA levels of *TTF-1*, *TTF-2*, *PAX-8* and *TSHR* by 60–85% compared to control treatment, co-treatment with RSV and TM increased mRNA levels of *TTF-1*, *TTF-2*, *PAX-8* and *TSHR* by 30–50% compared to treatment with TM alone. At the protein level, treatment with RSV alone increased TTF-1, TTF-2, PAX-8 and TSHR expression by 55–80% compared to control treatment (Figure 5B). Protein levels of TTF-1 and PAX-8 were decreased by 60–70% by treatment with TM alone compared to control treatment but increased by 70–90% by co-treatment with RSV and TM compared to treatment with TM alone. Protein levels of TTF-2 and TSHR in cells treated with TM alone and in cells co-treated with RSV and TM did not differ from control cells.

### 2.5. RSV Does Not Modulate TM-Induced Induction of Cytoprotective Genes but Decreases ROS Production in FRTL-5 Thyrocytes

**Figure 6:**
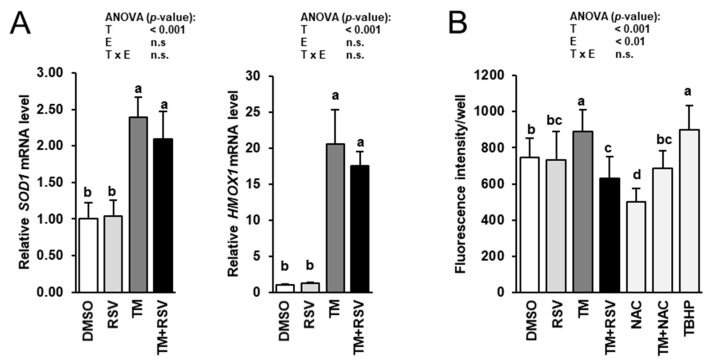
Effect of resveratrol (RSV), tunicamycin (TM) and TM and RSV on expression of cytoprotective genes (**A**) and intracellular ROS production (**B**) in FRTL-5 cells. After reaching confluence, cells were treated with either 0.1% DMSO (control), 10 µM RSV, 0.1 µg/mL TM, 10 µM RSV and 0.1 µg/mL TM, 1 mM NAC, 1 mM NAC and 0.1 µg/mL TM or 10 µM TBHP in 6H medium for 48 h. Relative mRNA levels of *SOD1* and *HMOX1* are expressed as fold of control. Data from qPCR and ROS production assay are means and SD calculated from three and five replicates, respectively, for the same treatment of three independent experiments and were subjected to 2-factorial (T = treatment, E = experiment) ANOVA. Bars without the same letters (^a,b,c,d^) differ (*p* < 0.05).

To study whether cytoprotective and antioxidant effects of RSV are involved in the observed inhibitory effect of RSV on TM-induced impairment of genes involved in thyroid hormone synthesis, the mRNA levels of two cytoprotective genes, superoxide dismutase 1 (*SOD1*) and hemeoxygenase 1 (*HMOX1*), and intracellular ROS production were determined in FRTL-5 cells treated with RSV in the absence and presence of TM for 48 h. The mRNA levels of *SOD1* and *HMOX1* were elevated by incubation of FRTL-5 cells with TM compared to vehicle control cells (Figure 6A). Incubation with RSV alone had no effect on the mRNA levels of *SOD1* and *HMOX1*. Combined incubation of FRTL-5 cells with RSV and TM did not alter mRNA levels of *SOD1* and *HMOX1* compared to FRTL-5 cells incubated with TM alone. Intracellular ROS production was increased by incubation with TM, whereas combined incubation with RSV and TM, but not RSV alone, decreased ROS production in FRTL-5 cells compared to vehicle control cells (Figure 6B). While incubation with 1 mM of the antioxidant NAC decreased ROS production, incubation with 10 µM of the exogenous inducer of oxidative stress TBHP stimulated ROS production compared to vehicle control cells (Figure 6B). In FRTL-5 cells co-treated with NAC and TM, ROS production did not differ from vehicle control cells. Both, NAC and TBHP did not impair cell viability as assessed by the MTT assay at the concentrations tested (data not shown).

## 3. Discussion

Recently, we have demonstrated that expression of genes involved in thyroid hormone synthesis and their key transcriptional regulators as well as iodide uptake are markedly impaired in FRTL-5 thyrocytes under conditions of ER stress [13]. This suggested a mechanistic link between ER stress and development of thyroid disorders associated with hypothyroidism [7,8,9,10,11] and indicated therapeutic options for the treatment of such thyroid disorders. In view of this, the present study revealed the following two key findings: First, RSV alleviates TM-induced ER stress in FRTL-5 thyrocytes. This was evident from an attenuated induction of ER stress-sensitive genes (HSPA5, DDIT3) and reduced splicing of XBP-1 in thyrocytes co-treated with RSV and TM as compared to thyrocytes treated with ER stress-inducer TM alone. The observed attenuation of TM-induced ER stress by RSV in the non-transformed FRTL-5 cell line is in line with a large number of published studies reporting an inhibitory effect of RSV on ER stress in different cell lines and tissues [19,20,21,22,23,24]. Second, the strong impairment of expression of genes involved in thyroid hormone synthesis and some of their transcriptional regulators (TTF-1, PAX-8) in FRTL-5 thyrocytes exposed to TM-induced ER stress is attenuated, but not completely abolished by RSV. Albeit direct proof is still pending, these results suggest that RSV stimulates thyroid hormone synthesis during TM-induced ER stress by attenuating the inhibitory effect of ER stress on transcription of both, thyroid hormone synthesizing genes and their regulators. The induction of NIS, TPO and TG and the increased iodide uptake in FRTL-5 cells treated with RSV, which was observed in the absence and presence of ER stress, is in line with a recent study demonstrating increased protein expression of NIS and increased iodide uptake in FRTL-5 cells treated with RSV [25]. However, our results are in sharp contrast to those reported from two recent studies, in which RSV (10 µM) was found to decrease mRNA and protein levels of NIS, TPO, TG, TTF-1 and PAX-8 in FRTL-5 thyrocytes in the absence of ER stress [27,28]. Although the reason for these opposing results cannot be definitely explained, differences in the experimental settings are likely causative. One important difference to our study is that the authors of the above-mentioned study [27,28] switched their cultured FRTL-5 cells to a TSH-depleted medium for 6 days to become quiescent prior to incubation with TSH for 24 h followed by incubation with RSV in the presence of TSH. By contrast, in our study FRTL-5 cells were treated with RSV in the presence of human pituitary-derived TSH (1 mU/mL) directly after reaching confluence without a period of TSH depletion. Despite the human TSH concentration used in the FRTL-5 cell culture medium was higher than the plasma TSH concentration in rats (approx. 1 µM/mL, [29,30]) due to species differences in the bioactivity of TSH, complete TSH depletion of thyrocytes does not reflect the physiological situation at all. It is well known that FRTL-5 cells depleted of TSH cease growth and alter cell morphology, while sudden addition of TSH rapidly stimulates growth and proliferation [31]. Despite experimental proof being missing, it is not unlikely that RSV exerts a quite different biological response in either TSH-preconditioned FRTL-5 cells or TSH-deprived FRTL-5 cells which are suddenly treated together with a potent cell proliferator.

Apart from the above-described key findings, our study shows that RSV attenuates deglycosylation of NIS and TG in thyrocytes induced by the action of TM. TM causes a general blockade of *N*-linked glycosylation of proteins, which primarily occurs in the ER, thereby impairing protein function [12,32,33]. In line with this, it has been shown that the function of NIS and TG, both of which are highly glycosylated proteins in their mature form, are glycosylation-dependent [6,34]. In the case of NIS, it has been demonstrated that glycosylation is a prerequisite for proper membrane localization of NIS and efficient iodide uptake across the plasma membrane [35], whereas TM-induced ER stress decreases membrane localization of NIS and iodide uptake [34]. Thus, our observations suggest that RSV improves thyrocyte function under conditions of TM-induced ER stress by attenuating the pronounced inhibition of both, transcription of key genes involved in thyroid hormone synthesis and their regulators and protein glycosylation in the ER. Whether or not RSV is generally able to improve thyrocyte function under conditions of ER stress remains to be demonstrated in future experiments using other ER stress inducers, such as thapsigargin. This, however, is not unlikely considering that RSV has been demonstrated to inhibit thapsigargin-induced ER stress in a human hepatocyte cell line [36].

It can be assumed that the antioxidant effects of RSV largely explain the observed inhibitory effect on TM-induced ER stress, because antioxidants are well-known to reduce ER stress via decreased production of ROS being potent stimuli of ER stress [37]. Previous studies have shown that RSV reduces ROS production via directly scavenging different ROS species [38,39] and/or modulating the expression and activity of antioxidant and cytoprotective genes, such as HMOX1 and SOD1, through activation of redox-sensitive transcription factors, such as nuclear factor erythroid 2-related factor 2 (Nrf2) [40,41]. Nrf2 is well known to be stimulated upon ER stress via activation of PERK as a cellular adaptive response to resolve ER stress [42]. In the present study, we found that RSV, like the potent antioxidant NAC, significantly decreases ROS production in FRTL-5 cells under conditions of TM-induced ER stress, while the strong TM-induced upregulation of Nrf2 target genes, such as SOD1 and HMOX1, was only partially (not significantly) reduced by RSV. Despite this insignificant modulation of TM-induced cytoprotective gene expression by RSV in FRTL-5 cells, the data from intracellular ROS production assay clearly show that RSV decreases ROS generation under conditions of TM-induced ER stress. Of note is that RSV has also been demonstrated to exert cytoprotective effects, such as antioxidant and anti-inflammatory effects, through the activation of SIRT1, which is a critical regulator of a wide variety of cellular responses including inflammation, stress resistance, mitochondrial biogenesis and apoptosis [43]. Several studies revealed that RSV activates SIRT1 both, directly and indirectly via AMPK activation [44,45,46,47]. In this context, it is worth mentioning that evidence from an in vivo-study with rats has been gained that RSV also exerts a positive effect on thyroid function by stimulating TSH secretion via the hypothalamic-pituitary axis and increases iodide trapping in the thyroid and triiodothyronine levels in plasma [48]. Interestingly, the authors of this study postulated that RSV exerts this effect indirectly via activation of SIRT1, as proposed for other polyphenolic compounds, such as kaempferol [49].

Despite showing that RSV exerts partial protection against TM-induced ER stress and attenuates the inhibitory effects of ER stress on gene expression in thyrocytes at the concentration used in the present study, it has to be mentioned that the plasma concentration of RSV in humans is substantially lower. The low plasma RSV concentration in humans is largely explained by extensive metabolism of RSV in the intestine and liver into RSV glucuronides and sulfates resulting in an oral RSV bioavailability of <1% [50]. In addition, colonic bacterial metabolism of RSV into different metabolites, such as dihydro-RSV and lunularin, contributes to the low RSV bioavailability. In view of this, future studies using RSV analogues with improved bioavailability, such as methylated RSV derivatives, should clarify if RSV analogues also provide partial protection against ER stress induction in thyrocytes and may come into question as therapeutic option for the treatment of specific thyroid disorders associated with hypothyroidism [7,8,9,10,11].

In conclusion, the present study shows that RSV alleviates TM-induced ER stress and attenuates the strong impairment of expression of genes involved in thyroid hormone synthesis and their transcriptional regulators in FRTL-5 thyrocytes exposed to ER stress. Thus, RSV may be useful for the treatment of specific thyroid disorders, provided that strategies with improved oral bioavailability of RSV are applied.

## 4. Materials and Methods 

### 4.1. Cell Culture

The FRTL-5 cell line was obtained from Cell Line Service (Eppelheim, Germany). Cells were cultured in Ham’s F12 medium supplemented with 5% newborn calf serum, 1% antibiotic-antimycotic-mixture and a six-hormone mixture (6H medium containing 1 mU/mL human TSH, 10 μg/mL insulin, 10 pmol/mL hydrocortisone, 5 μg/mL transferrin, 10 ng/mL somatostatin, and 10 ng/mL glycyl-l-histidyl-l-lysine acetate; all from Sigma-Aldrich, Taufkirchen, Germany) at 37 °C in a humidified atmosphere composed of 95% air and 5% CO_2_, as described recently [51]. The medium was changed every 2 days. After reaching a confluence of 80%, the cells were either sub-cultivated or used for experiments. All experiments were performed at least two times from a different cell passage number (=independent experiments). An independent experiment was defined as an experiment performed with cells of a specific passage number and included seeding, treatment and analysis.

### 4.2. Cell Viability Assay

For evaluation of cell viability, FRTL-5 cells were seeded in 96-well culture plates at a density of 8 × 10^4^ cells/well. After reaching confluence, cells were treated with increasing concentrations of either TM (Sigma-Aldrich, Taufkirchen, Germany; dissolved in DMSO), RSV (Sigma-Aldrich, Taufkirchen, Germany; dissolved in DMSO), *N*-acetyl l-cysteine (NAC) (Sigma-Aldrich; dissolved in H_2_O), tert-butyl-hydrogen-peroxide (TBHP) (Abcam, Cambridge, UK), or the combination of TM and RSV and of TM and NAC in 6H medium for 6 or 48 h. Control cells were treated with the same vehicle concentration (0.1% DMSO). The 3-(4,5-dimethylthiazol-2-yl)-2,5-diphenyltetrazolium bromide (MTT) assay was used to assess cell viability as described recently [13]. Cell viabilities are expressed as percentage of vehicle control cells (viability of vehicle control cells was set to 100%).

### 4.3. RNA Isolation and qPCR Analysis

For qPCR experiments, FRTL-5 cells were seeded in 24-well culture plates at a density of 3 × 10^5^ cells/well. After reaching confluence, cells were treated with either 10 µM RSV, 0.1 µg/mL TM or 10 µM RSV combined with 0.1 µg/mL TM or 0.1% DMSO as vehicle control in 6H medium for 6 or 48 h. Following treatment, total RNA was extracted from cells using Trizol reagent (Invitrogen, Karlsruhe, Germany) according to the manufacturer’s protocol. The synthesis of cDNA and qPCR analysis were performed as described recently in detail [52,53,54]. Characteristics of gene-specific primers synthesized by Eurofins MWG Operon (Ebersberg, Germany) are listed in Table 1. Normalization was carried out by geometric averaging of multiple reference genes (here: *ACTB*, *RPL13*, *TOP*, *YWHAZ*; *M*-values between 0.242 and 0.364; V-values V3/V4 between 0.090 and 0.146) as described by [55]. Normalized mRNA levels are expressed as fold of vehicle control cells (=1.0). 

### 4.4. Protein Separation and Immunoblotting

For western blot experiments, FRTL-5 cells were seeded in 6-well culture plates at a density of 8 × 10^5^ cells/well. After reaching confluence, cells were treated as described for qPCR experiments. For detection of HSPA5, DDIT3, TTF-1, TTF-2, PAX-8, TSHR, NIS and TG, preparation of cell lysates, determination of protein concentration of cell lysates and separation of proteins by SDS-PAGE were carried out as described recently [13]. The blots were incubated with the following primary antibodies at 4 °C overnight: rabbit anti-HSPA5 (1:5000), mouse anti-DDIT3 (1:2000), mouse anti-NIS against deglycosylated NIS (1:500) (all from Thermo Fisher Scientific, Darmstadt, Germany), rabbit anti-NIS against both glycosylated and deglycosylated NIS (1:2000) (kindly provided from Prof. Nancy Carrasco, Department of Cellular and Molecular Physiology, Yale University School of Medicine, New Haven, CT, USA), mouse anti-TTF-1 (1:500), mouse anti-TTF-2 (1:500), mouse anti-PAX-8 (1:500), rabbit anti-TSHR (1:1000) (all from Santa Cruz Biotechnology, Heidelberg, Germany), rabbit anti-TG against both glycosylated and deglycosylated TG (1:5000; Abcam, Cambridge, UK), and either mouse anti-β-actin (1:20,000; Abcam) or rabbit anti-vinculin (1:10,000; Thermo Fisher Scientific, Darmstadt, Germany) as reference proteins for normalization at RT for 2 h. After washing, the blots were incubated with the following secondary antibodies—anti-rabbit-IgG (1:10,000; Sigma-Aldrich, Taufkirchen, Germany) or anti-mouse IgG antibody (1:10,000; Santa Cruz Biotechnology, Heidelberg, Germany) at RT for 2 h. Blots were developed using ECL Plus (GE Healthcare, München, Germany) and band intensities were evaluated densitometrically as described recently [53]. Normalized protein levels are expressed as fold of vehicle control cells (=1.0).

### 4.5. Analysis of NIS and TG Deglycosylation

Peptide-N-Glycosidase F (PNGase F) from New England BioLabs (Frankfurt/Main, Germany) was used to analyse the deglycosylation of NIS and TG protein according to the manufacturer’s protocol. Briefly, 15 µg of total protein was denatured at 100 °C for 10 min. 1 µL of PNGase F (500,000 U/mL) was added and incubated at 37 °C for 60 min. Following SDS-PAGE of PNGase F-treated and untreated samples, immunoblotting was carried out as described above.

### 4.6. Nonradioactive Iodide Uptake Assay

For measuring iodide uptake, FRTL-5 cells were seeded in 96-well plates at a density of 8 × 10^4^ cells/well. After reaching confluence, cells were treated identical as for qPCR and immunoblotting experiments. Iodide uptake was measured using the nonradioactive iodide uptake assay from Waltz et al. [56]. In brief, cells were incubated in uptake buffer with 10 µM NaI in the absence or presence of KClO_4_, a competitive inhibitor of NIS, at 20 °C for 60 min. The supernatants were discarded, and cell-trapped iodide was determined using the As/Ce method which is based on the specific reduction of Ce(IV) to Ce(III) catalysed by iodide in the presence of arsenious acid, a reaction which causes a change from yellow to colourless in proportion to the iodide concentration. The cell-trapped iodide concentration measured in the presence of KClO_4_ corresponds to the level of iodide taken up when NIS is totally inhibited (passive iodide uptake), while that measured in the absence of KClO_4_ corresponds to total iodide uptake (passive and NIS-mediated iodide uptake). The cell-trapped iodide concentration of cell lysates was determined using a calibration curve prepared from known NaI standards. Absorbance measurement at 420 nm was carried out using an Infinite M200 microplate reader (Tecan, Mainz, Germany). The cell-trapped iodine concentration was normalized by the protein concentration of cell lysates determined by the BCA assays as described for immunoblotting.

### 4.7. Intracellular ROS Production Assay

Intracellular ROS levels were measured using DCFDA/H2DCFDA Cellular ROS Assay Kit (Abcam, Cambridge, UK) according to the manufacturer´s protocol. FRTL-5 cells were seeded in 96-well plates at a density of 8 × 10^4^ cells/well. After reaching confluence, cells were treated with either 0.1% DMSO as vehicle control, 10 µM RSV, 0.1 µg/mL TM, 10 µM RSV combined with 0.1 µg/mL TM, 1 mM NAC, 1 mM NAC combined with 0.1 µg/mL TM or 10 µM TBHP as positive control for ROS generation in 6H medium for 48 h. Following treatment, the cells were incubated with 20 µM of labeled or unlabeled (as blank) 2′,7′-dichlorofluorescein diacetate (DCFDA) at 37 °C for 45 min. The fluorescence intensity was measured using an Infinite M200 microplate reader (Tecan, Mainz, Germany) at excitation and emission wavelengths of 485 and 535 nm, respectively. Results are shown as fluorescence intensity per well.

### 4.8. Statistical Analysis

Statistical analysis was performed using the Minitab statistical software (Rel. 13.0, State College, PA, USA). Data from qPCR and ROS production assay are means and SD calculated from three and five replicates, respectively, for the same treatment of three independent experiments. Data from qPCR and ROS production assay were subjected to 2-factorial ANOVA with classification factors being treatment (T), experiment (E) and the interaction of both factors (T × E). Data from immunoblotting are means and SD calculated from one replicate for the same treatment of three independent experiments. Data from iodide uptake assay and MTT assay are means and SD from one independent experiment performed in triplicate and octuplicate, respectively. Iodide uptake assay and MTT assay were performed twice and both independent experiments showed similar results. Data from immunoblotting, iodide uptake assay and MTT assay were analysed by 1-factorial ANOVA. For statistically significant *F* values, individual means of the treatment groups were compared by Fisher’s multiple range test. Effects were considered significant if *p* < 0.05.

## Figures and Tables

**Table 1:** Characteristics of the gene-specific primers used for qPCR analysis.

Gene Name	Primer Sequence (Forward, Reverse)	Product Size (bp)	NCBI GenBank
*Reference Genes*			
*ACTB*	GACCTCTATGCCAACACAGT CACCAATCCACACAGAGTAC	154	NM_031144
*RPL13*	CTTAAATTGGCCACGCAGCT CTTCTCAACGTCTTGCTCTG	198	XR_086310
*TOP1*	GAAGAACGCTATCCAGAAGG GCTTTGGGACTCAGCTTCAT	137	NM_022615
*YWHAZ*	GACGGAAGGTGCTGAGAAA GCAGCAACCTCAGCCAAGT	198	NM_013011
*Target Genes*			
*DDIT3*	ACAAGCACCTCCCAAAGCCC TGCTCCTTCTCCTTCATGCGC	155	NM_001109986
*HMOX1*	AGCATGTCCCAGGATTTGTC TCACCAGCTTAAAGCCTTCC	130	NM_012580
*HSAP5*	TCAGCCCACCGTAACAATCAAGG TCCTCAGCAAACTTCTCGGCG	282	NM_013083
*NIS*	GCTGTGGCATTGTCATGTTC TGAGGTCTTCCACAGTCACA	219	NM_052983
*PAX-8*	CCTTACTCAACAGTACCCTGG AGCTAGAACTGGAGAGCTCTG	162	NM_031141
*SOD1*	TATGGTGGTCCACGAGAAAC AATCACACCACAAGCCAAGC	100	NM_017050
*TG*	GTTCCTACGTGTACTAGTGAG CATACTGGAGTTGGAGAGCAG	196	NM_030988
*TPO*	CAGGTGTTGAGAAGCAGTTG CTTTGAAAGCTGTAGCCAGG	255	NM_019353
*TSHR*	CCAGAAGCTTGACTTACATAG CATGTAAGGGTTGTCTGTGAT	161	NM_012888
*TTF-1*	GCATGAATATGAGCGGCATGG ACTTCTGCTGCTTGAAGCGTC	153	NM_013093
*TTF-2*	GAAGTGGCAGAACAGCATCC AGCTGCCGCTCTCGAACATG	139	NM_138909
*XBP1*	GACACGCTTGGGGATGAATGC AGAGGCAACAGCGTCAGAATCC	192/166	NM_001004210 (XBP1) NM_001271731 (sXBP1)

## Data Availability

The data presented in this study are available in the article.
